# Desfechos Intra-Hospitalares no Registro Brasileiro de Implante de Bioprótese Aórtica por Cateter – 14 Anos em Análise

**DOI:** 10.36660/abc.20230551

**Published:** 2024-05-07

**Authors:** Maria Cristina Meira Ferreira, Viviana de Mello Guzzo Lemke, Maria Sanali Moura de Oliveira Paiva, Emilia Matos do Nascimento, Basílio de Bragança Pereira, Gláucia Maria Moraes de Oliveira

**Affiliations:** 1 Universidade Federal do Rio de Janeiro Rio de Janeiro RJ Brasil Universidade Federal do Rio de Janeiro, Rio de Janeiro, RJ – Brasil; 2 Cardiocare Clinica Cardiológica Curitiba PR Brasil Cardiocare Clinica Cardiológica, Curitiba, PR – Brasil; 3 Instituto Atena de Pesquisa Clínica Natal RN Brasil Instituto Atena de Pesquisa Clínica, Natal, RN – Brasil; 4 Universidade do Estado do Rio de Janeiro Rio de Janeiro RJ Brasil Universidade do Estado do Rio de Janeiro, Rio de Janeiro, RJ – Brasil

**Keywords:** Valvopatia Aórtica, Estenose da Valva Aórtica, Implante de Prótese de Valva Cardíaca, Substituição da Valva Aórtica Transcateter, Próteses Valvulares Cardíacas

## Abstract

**Fundamento:**

O implante valvar aórtico transcateter (TAVI) apresenta crescimento exponencial de suas indicações e foi incorporado ao Sistema Único de Saúde em 2022, sendo necessário avaliar seu uso no Brasil.

**Objetivo:**

Conhecer os fatores associados a mortalidade e complicações não fatais intra-hospitalares, em ambos os gêneros, na população do Registro Brasileiro de Implante de Bioprótese Aórtica por Cateter e Novas Tecnologias (RIBAC-NT).

**Método:**

Análise do banco de dados RIBAC-NT de 2008 a 2022. Aplicados modelos logísticos e *machine learning* na avaliação estatística da associação das variáveis com os desfechos, empregando o *software R* e nível de significância de 5%.

**Resultados:**

Analisados 2.588 pacientes (mulheres, 51,2%; óbito intra-hospitalar, 8,2%). Mortalidade associou-se a complicações do procedimento, dentre elas destacam-se complicações vasculares (CV) maiores e insuficiência renal aguda (IRA) (p< 0,001). A CV maior ocorreu em 6%, com 34% de mortalidade; IRA ocorreu em 8,8%, com 13% de mortalidade, que aumentou até 8 vezes quando IRA coexistiu com outras complicações. Complicações não fatais ocorreram em 50,5% do total de pacientes, acometendo 63% daqueles com bioprótese de 1^a^ geração (1G) e 39% daqueles com bioprótese de 2^a^ geração (2G) p<0,001. O acesso não femoral e o ritmo cardíaco influenciaram as complicações não fatais nas próteses 1G, enquanto complicações das próteses 2G associaram-se ao gênero feminino (39,6% *vs.* 30,4%, p= 0,003).

**Conclusão:**

A mortalidade intra-hospitalar na população do RIBAC-NT associou-se diretamente a complicações do procedimento, principalmente CV maior e IRA. A ocorrência de complicações não fatais diferiu conforme o gênero e o tipo da bioprótese.

**Figure:**
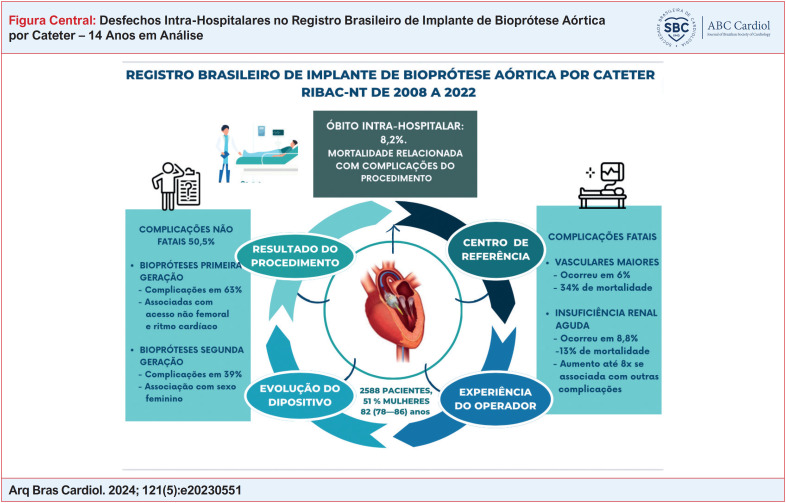


## Introdução

A estenose aórtica degenerativa afeta atualmente 2% a 5% dos adultos com mais de 65 anos, tendo se tornado um problema de saúde pública mundial nas últimas décadas.^1^ No Brasil, a prevalência de doença valvar aórtica calcificada aumentou 201,8% de 1990 para 2019, refletindo o envelhecimento da população.^2^ O implante valvar aórtico transcateter (TAVI) surgiu como opção para reduzir a mortalidade dos pacientes portadores de estenose aórtica inoperáveis e de alto risco cirúrgico.^3 - 5^ Embora diversos estudos clínicos tenham tido importante papel no avanço da TAVI, publicação recente de Barili et al.,^6^ com análise de 8 estudos randomizados de TAVI *versus* cirurgia de troca valvar aórtica, identificou a ocorrência de diversos vieses com desbalanceamentos favoráveis a TAVI, como desvio da randomização 6,7 vezes e perda de seguimento 2,5 vezes menores no grupo TAVI, enquanto tratamentos adicionais durante o procedimento foram 3,7 vezes mais frequentes no grupo cirúrgico.

Objetivando entender o emprego da TAVI, vários registros realizados em diversos países mostraram mortalidade e complicações não fatais com taxas mais elevadas que nos estudos clínicos.^7^ Com intuito de conhecer os resultados da incorporação dessa tecnologia no Brasil, a Sociedade Brasileira de Hemodinâmica e Cardiologia Intervencionista (SBHCI) implementou um registro dos pacientes submetidos a TAVI no território nacional denominado Registro Brasileiro de Implante de Bioprótese Aórtica por Cateter e Novas Tecnologias (RIBAC-NT), que reúne dados dos pacientes submetidos a esse procedimento desde 2008.

Com os esforços desenvolvidos para incorporação da TAVI pelo Sistema Único de Saúde (SUS) nos últimos anos, é importante avaliar seu desempenho nos diversos cenários clínicos no Brasil no que tange às complicações decorrentes do procedimento. Um problema particular refere-se às mulheres, que têm maior expectativa de vida e, portanto, maior probabilidade de serem submetidas a TAVI, além de mais complicações decorrentes do procedimento, ainda que sem maior mortalidade.^8 - 10^ Desse modo, o objetivo deste estudo é avaliar os fatores associados aos desfechos mortalidade e complicações não fatais intra-hospitalares nos pacientes do RIBAC-NT submetidos a TAVI, além de avaliar se existe associação do gênero com esses desfechos.

## Material e Métodos

Estudo observacional, retrospectivo, de análise do banco de dados do RIBAC-NT, um registro de participação voluntária e que inclui 266 centros em 20 unidades federativas brasileiras, no período de janeiro de 2008 a janeiro de 2022. O estudo foi aprovado pelo Comitê de Ética e Pesquisa em 21/9/2022 (CAAE 60808622.4.0000.5252).

As biopróteses utilizadas no RIBAC-NT foram classificadas conforme a geração de fabricação e o mecanismo de expansão ( Tabela 1 suplementar ).

As complicações decorrentes do procedimento foram padronizadas de acordo com o documento *Valve Academic Research Consortium* 2012 (VARC-2).^11^ Neste estudo, as complicações hemorrágicas classificadas no VARC-2 como maior e de risco de vida foram agrupadas sob a denominação ‘complicações hemorrágicas maiores’.

### Análise estatística

As características da população foram descritas com variáveis qualitativas dicotômicas, representadas como percentual de ocorrência, e com variáveis contínuas, representadas como mediana e intervalo interquartil. Na análise comparativa entre percentuais de ocorrência de eventos, aplicou-se o teste do qui-quadrado. O teste de normalidade de Shapiro-Wilk rejeitou a hipótese de normalidade das variáveis. A não normalidade dos dados foi confirmada através de inspeção gráfica.

Para análise dos fatores associados aos desfechos mortalidade e complicações não fatais, consideraram-se as variáveis demográficas, clínico-laboratoriais e as comorbidades, assim como os dados técnicos e as complicações do procedimento.

Inicialmente, empregou-se o modelo de regressão logística com o uso da regularização *elastic net* ,^12^ método para seleção prévia das variáveis independentes. Em seguida, outros modelos logísticos foram utilizados, descartando-se as variáveis que não apresentaram significância estatística no modelo anterior. O uso de diversos modelos objetivou identificar padrões e associações que podem não ser facilmente detectadas em apenas um modelo devido à complexidade dos dados.

Técnicas de *machine learning* foram empregadas com os modelos não paramétricos baseados em árvores de classificação.^13^ Uma estrutura hierárquica baseada em algoritmos foi formada, buscando-se associações e interdependência das variáveis entre os nós.

O modelo multinomial foi utilizado para análise dos desfechos compostos de morte e complicações não fatais.

O pacote Partykit do *software* R foi empregado para análise.^14 , 15^ O nível de significância estatística utilizado foi de 5%.

Realizou-se uma análise temporal do desfecho mortalidade e as complicações associadas nos anos em estudo, utilizando-se o *star plot* , técnica de visualização de dados para análise multivariada. Esse método produz gráficos em forma de estrela, onde cada variável é representada por uma área proporcional ao valor observado. Os dados utilizados nessa análise foram os resultantes da divisão dos valores correspondentes às variáveis de maior impacto nos desfechos nos anos de 2008 a 2021.^16^

## Resultados

**Figura 1:**
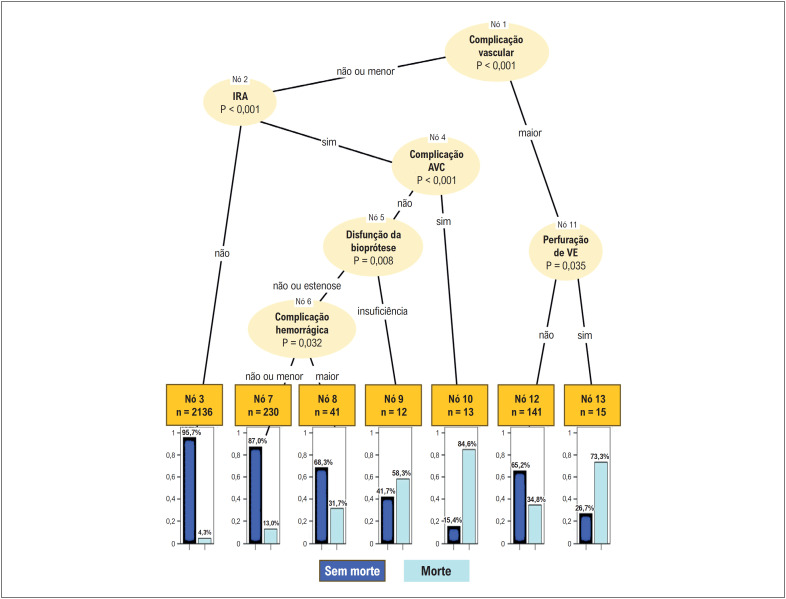
Árvore de classificação dos fatores associados a mortalidade intra-hospitalar. IRA: insuficiência renal aguda; AVC: acidente vascular cerebral; VE: ventrículo esquerdo.

Procedimento de TAVI foi realizado em 3.793 pacientes (1883 homens e 1910 mulheres), dos quais 1.205 foram excluídos da análise por falta de algumas informações (619 homens e 586 mulheres, p= 0,067). Não houve diferença estatisticamente significativa entre os gêneros. Dados completos foram obtidos em 2.588 pacientes, que foram submetidos à análise ( Figura 1 suplementar ).

Óbito intra-hospitalar ocorreu em 8,2% dos pacientes, sendo percentualmente mais elevado nas mulheres. Os pacientes que morreram eram mais idosos, com a maioria da população do estudo em classe funcional III e IV da *New York Heart Association* . Dentre as comorbidades, a doença pulmonar obstrutiva crônica (DPOC) apresentou maior percentual de ocorrência nos que morreram. A depuração de creatinina foi mais elevada nos sobreviventes (p= 0,0001) e o escore *Society of Thoracic Surgeons* (STS) foi mais elevado nos que morreram (p<0,0001) ( Tabela 1 ).

**Tabela 1:** Taxas de ocorrência das variáveis demográficas e clínico-laboratoriais e das comorbidades nos pacientes do RIBAC-NT, comparativo entre sobreviventes e óbito

		Total	Sobreviventes	Óbito
**Número de pacientes, n (%)**		2.588 (100%)	2.375 (91,8%)	213 (8,2%)
**Dados demográficos**				
	Homens, n (%)	1.264 (48,8%)	1.181 (93,4%)	83 (6,6%)
	Mulheres, n (%)	1.324 (51,2%)	1.194(90,2%)	130 (9,8%)
	Idade, mediana (IQ)	82 (78—86)	82 (77—86)	84 (79—88)
	IMC, kg/m^2^, mediana (IQ)	25,8 (23,3—29,0)	25,8 (23,3—29)	26,2 (23,1—29,4)
**Dados clínicos**				
	Classe funcional III ou IV, n (%)	1924 (74,3%)	495 (88,6%)	64 (11,4%)
	Angina pectoris, n (%)	637 (24,6%)	594 (93,2%)	43 (6,8%)
	Síncope, n (%)	681 (26,3%)	631 (92,7%)	50 (7,3%)
**Comorbidades**				
	DAC, n (%)	1.399 (54%)	1.284 (91,8%)	115 (8,2%)
	IAM prévio, n (%)	363 (14%)	332 (91,5%)	31 (8,5%)
	AVC prévio, n (%)	207 (7,9%)	188 (90,8%)	19 (9,2%)
	DC e cerebrovascular, n (%)	421 (16,2%)	383 (91,0%)	38 (9,0%)
	DPOC, n (%)	474 (18,3%)	418 (88,2%)	56 (11,8%)
	Aneurisma de aorta, n (%)	108 (4%)	99 (91,7%)	9 (8,3%)
	Diabetes mellitus, n (%)	869 (33,6%)	800 (92,1%)	69 (7,9%)
	Dislipidemia, n (%)	1.495 (57,8%)	1.375(92,0%)	120 (8,0%)
	HAS, n (%)	2.148 (82,9%)	1.966 (91,5%)	182 (8,5%)
	DAP, n (%)	493 (19%)	448 (90,9%)	45 (9,1%)
	HAP, n (%)	607 (23,5%)	547 (90,1%)	60 (9,9%)
	IRC, n (%)	1.917 (74%)	1.748 (91,2%)	169 (8,8%)
**Dados laboratoriais**				
	Ritmo sinusal, n (%)	2.043 (78,9%)	1.883 (92,2%)	160 (7,8%)
	Ritmo de marca-passo, n (%)	214 (8,3%)	199 (93,0%)	15 (7,0%)
	Fibrilação/Flutter atrial, n (%)	331 (12,8%)	293 (88,5%)	38 (11,5%)
	Creatinina (mg/dl), mediana (IQ)	1,1 (0,9—1,4)	1,1 (0,9—1,4)	1,1 (0,9—1,6)
	Depuração creatinina (ml/min), mediana (IQ)	46,6 (34,8—62,2)	47 (35,2—62,4)	39,5 (29,8—57,4)
	FE Ecocardiograma, (%), mediana (IQ)	63 (53—69)	63 (53—69)	63 (50—69)
	Escore STS, mediana (IQ)	5,5 (3,4—10,2)	5,3 (3,3—9,9)	7,7 (4,5—14,0)

AVC: acidente vascular cerebral; DAC: doença arterial coronariana; DAP: doença arterial periférica; DC: doença carotídea; DPOC: doença pulmonar obstrutiva crônica; FE: fração de ejeção; HAP: hipertensão arterial pulmonar; HAS: hipertensão arterial sistêmica; IAM: infarto agudo do miocárdio; IMC: índice de massa corporal; IQ: intervalo interquartil; IRC: insuficiência renal crônica; STS: Society of Thoracic Surgeons.

O modelo logístico identificou as variáveis DPOC, nível de creatinina e complicações do procedimento, sejam clínicas, cirúrgicas ou mecânicas decorrentes da bioprótese, como as associadas com morte ( Tabela 2 ).

**Tabela 2:** Variáveis identificadas pelo modelo de regressão logística como associadas ao desfecho óbito

	Estimate	Std. Error	z value	Pr(>|z|)	
Creatinina	0.25226	0.07389	3.414	0.000640	***
DPOC	0.67307	0.18777	3.585	0.000338	***
Complicação IAM	2.03395	0.53088	3.831	0.000127	***
Complicação AVC	1.81760	0.32690	5.560	2.70e-08	***
Complicação hemorrágica maior	0.91282	0.23560	3.874	0.000107	***
Complicação hemorrágica menor	-0.04292	0.51031	0.084	0.932966	
Insuficiência renal aguda	1.40844	0.17848	7.891	2.99e-15	***
Complicação vascular maior	1.25127	0.26325	4.753	2.00e-06	***
Complicação vascular menor	-0.50648	0.49991	-1.013	0.310990	
Estenose de bioprótese	12.15345	535.41122	-0.023	0.981890	
Insuficiência de bioprótese	1.34886	0.41188	3.275	0.001057	**
Bloqueio de ramo esquerdo	-0.58469	0.26618	-2.197	0.028047	*
Necessidade de MPD	-0.93291	0.32887	-2.837	0.004558	**
Obstrução coronariana	3.77983	1.23676	3.056	0.002241	**
CIV	1.94111	1.14184	1.700	0.089135	
Perfuração de VE	2.09158	0.51046	4.097	4.18e-05	***

Significado estatístico

‘***’ 0.001,

‘**’ 0.01,

‘*’ 0.05,

‘ ’1. *Estimate* : estimativa; *Std. Error* : erro-padrão da estimativa; *z value* : percentil da distribuição normal padrão; Pr(>|z|): nível de significância observado. AVC: acidente vascular cerebral; CIV: comunicação interventricular; DPOC: doença pulmonar obstrutiva crônica; IAM: infarto agudo do miocárdio; MPD: marca-passo definitivo; VE: ventrículo esquerdo.

A árvore de classificação, construída a partir das variáveis selecionadas no modelo logístico, demonstrou a associação entre complicações do procedimento e morte. A primeira variável selecionada foi complicação vascular (CV) maior (nó 1), seguida de insuficiência renal aguda (IRA) (nó 2). Quando não ocorreu nenhuma dessas complicações, a mortalidade foi 4,3% (nó 3) ( Figura 1 ).

Pacientes que apresentaram CV maior associada com perfuração de ventrículo esquerdo (VE) apresentaram mortalidade de 73,3% (nós 1, 11 e 13) e, na ausência de perfuração de VE, a mortalidade foi 34,8% (nós 1 e 12). Quando a CV inexistiu ou foi do tipo menor e na presença de IRA, a mortalidade variou conforme coexistência com: acidente vascular cerebral (AVC), mortalidade de 84,6% (nós 2, 4 e 10); disfunção de bioprótese do tipo insuficiência, mortalidade de 58,3% (nós 2, 5 e 9); complicação hemorrágica maior, mortalidade de 31,7% (nós 2, 6 e 8); e complicação hemorrágica menor, mortalidade de 13% (nós 2, 6 e 7) ( Figura 1 ).

Os modelos estatísticos não identificaram gênero como fator associado a mortalidade, embora houvesse predomínio do gênero feminino nas complicações. A CV maior ocorreu em 156 (6%) pacientes, sendo mais frequente em mulheres (8,1% *vs* . 3,9%, p< 0,001), a perfuração de VE ocorreu em 25 (0,9%), sendo duas vezes mais frequente no gênero feminino (1,4% *vs* . 0,6%, p= 0,03) e a complicação hemorrágica menor ocorreu em 87 (3,7%), predominando em mulheres (5,3% *vs* . 3,2%, p=0,05). Observaram-se IRA em 355 (13,7%) pacientes e AVC em 65 (2,5%), ambos sem diferença entre os gêneros. A disfunção da bioprótese do tipo insuficiência ocorreu em 47 (1,8%) pacientes, com predomínio em homens (2,4% *vs.* 1,3%, p=0,03), e a do tipo estenose ocorreu em somente 1 paciente no estudo.

Ao avaliar as complicações não fatais, observou-se que 1.308 (50,5%) pacientes tiveram algum tipo de complicação relacionada ao procedimento. As complicações foram mais frequentes nas biopróteses de 1ª geração (1G), utilizadas em 47,3% da população, do que nas de 2ª geração (2G), tendo ocorrido em 774 (63,1%) pacientes *versus* 532 (39,1%), respectivamente, p<0,001.

Entre as variáveis independentes com associação de maior significado estatístico com as complicações não fatais, o modelo de regressão logística identificou gênero feminino, AVC e ritmo de marca-passo prévios, assim como tipo de bioprótese ( Tabela 3 ).

**Tabela 3:** Variáveis identificadas pelo modelo de regressão logística como associadas ao desfecho complicações não fatais

	Estimate	Std.Error	z value	Pr(>|z|)	
Mulheres	0.20839	0.08689	2.398	0.01647	*
História AVC	0.52861	0.16235	3.256	0.00113	**
Ritmo de MPD	-0.74091	0.16631	-4.455	8.39e-06	***
Ritmo de FA/Flutter atrial	-0.13150	0.13223	-0.99	0.32001	
Acesso vascular não femoral	0.38136	0.19996	1.907	0.05650	
Bioprótese autoexpansível	-0.64416	0.08935	-7.210	5.61e-13	***
Bioprótese ME	0.21087	0.28060	0.751	0.45236	
Bioprótese 2^a^ geração	-0.94258	0.08841	-10.661	< 2e-16	***

Significado estatístico

‘***’ 0.001,

‘**’ 0.01,

‘*’ 0.05, ‘ ’1. *Estimate* = estimativa, = erro-padrão da estimativa, *z value* = percentil da distribuição normal padrão, Pr(>|z|) = nível de significância observado. AVC: acidente vascular cerebral; FA: fibrilação atrial; MPD: marca-passo definitivo; ME: mecanicamente expansível.

**Figura 2:**
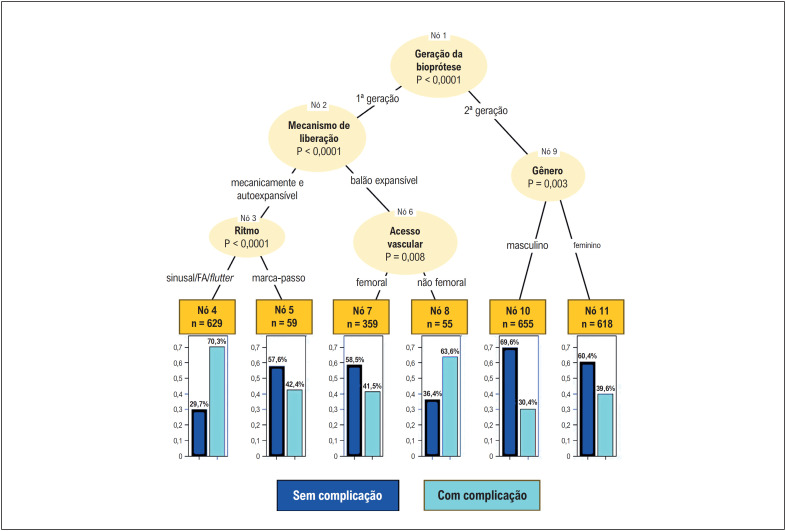
Árvore de classificação dos fatores associados a complicações não fatais intra-hospitalares.

A árvore de classificação, construída a partir das variáveis selecionadas no modelo logístico, mostrou que as complicações não fatais diferenciam-se conforme a geração de fabricação e o mecanismo de liberação da bioprótese utilizada. O nó 1 divide a árvore em dois grupos de acordo com a geração da bioprótese utilizada. As próteses 1G apresentam associação de complicações diferente conforme o mecanismo de liberação (nó 2). Quando autoexpansível (AE) ou mecanicamente expansível (ME), o percentual de complicação diferiu conforme o ritmo cardíaco (nó 3), tendo sido 70,3% em pacientes com ritmo sinusal ou fibrilação/ *flutter* atrial (nó 4) e 42,4% naqueles com ritmo de marca-passo (nó 5). As complicações das próteses 1G balão expansíveis (BE) apresentaram associação com o acesso vascular utilizado (nó 6). No acesso femoral, a taxa de complicação foi 41,5% (nó 7) e, no acesso não femoral, 63,6% (nó 8) ( Figura 2 ).

Quando próteses 2G foram empregadas, a ocorrência de complicações associou-se ao gênero (nó 9), tendo sido menor no masculino (30,4% - nó 10) do que no feminino (39,6% - nó 11) ( Figura 2 ).

O modelo multinomial aplicado na análise dos desfechos compostos de morte e complicações não fatais mostrou associações entre ritmo de marca-passo e complicações não fatais, entre DPOC e morte e entre o uso de próteses BE e complicações não fatais e fatais ( Tabela 4 ).

**Tabela 4:** Variáveis identificadas pelo modelo de regressão logística como associadas aos desfechos complicações não fatais e fatais

	Estimate	Std. Error	z value	Pr(>|z|)	
**Complicações não fatais**					
Creatinina	-0.071772	0.057691	-1.2441	0.2134779	
Angina	-0.120351	0.097514	-1.2342	0.2171300	
DPOC	0.010330	0.110159	0.0938	0.9252862	
Ritmo de marca-passo	-0.672786	0.161549	-4.1646	3.119e-05	***
Ritmo FA/ *flutter* atrial	-0.103293	0.128402	-0.8045	0.4211338	
Bioprótese BE	-0.668686	0.086679	-7.7145	1.221e-14	***
Bioprótese ME	0.047406	0.516099	0.0919	0.9268136	
**Complicações com MORTE**					
Creatinina	0.121578	0.072942	1.6668	0.0955582	
Angina	-0.357984	0.184781	-1.9373	0.0527033	
DPOC	0.495042	0.174086	2.8437	0.0044598	**
Ritmo de marca-passo	-0.395878	0.286658	-1.3810	0.1672751	
Ritmo de FA/ *flutter* atrial	0.298336	0.201967	1.4771	0.1396355	
Bioprótese BE	-0.523401	0.155061	-3.3755	0.0007369	***
Bioprótese ME	0.047406	0.516099	0.0919	0.9268136	

Significado estatístico:

‘***’ 0.001,

‘**’ 0.01,

‘*’ 0.05,

‘ ’1. *Estimate* = estimativa, *Std. Error* = erro-padrão da estimativa, *z value* = percentil da distribuição normal padrão, Pr(>|z|) = nível de significância observado. DPOC: doença pulmonar obstrutiva crônica; FA: fibrilação atrial; BE: balão expansível; ME: mecanicamente expansível.

**Figura 3:**
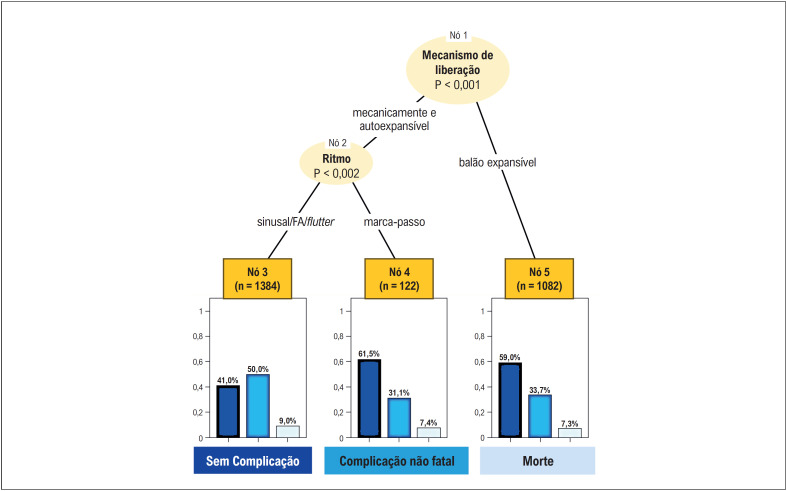
Árvore de classificação do desfecho composto morte e complicações não fatais intra-hospitalares.

A árvore de classificação construída a partir da seleção de variáveis do *elastic net* do modelo multinomial mostra que o mecanismo de liberação da prótese (nó 1) divide a árvore em dois ramos. Nos pacientes que receberam próteses AE e ME, as complicações variaram conforme o ritmo cardíaco prévio (nó 2). Com ritmo sinusal ou fibrilação/ *flutter* atrial, as complicações não fatais ocorreram em 50% dos pacientes e as fatais, em 9% (nó 3). Nos pacientes com ritmo de marca-passo prévio, as complicações não fatais ocorreram em 31,1% e as fatais, em 7,4% (nó 4). Esses dados reforçam a análise do modelo estatístico anterior. Nos pacientes que usaram próteses BE, as complicações não fatais ocorreram em 33,7% e as fatais, em 7,3% (nó 5), independentemente do ritmo cardíaco. Houve maior frequência de complicações não fatais nos pacientes do nó 3, porém, as taxas de mortalidade foram similares nos nós 3, 4 e 5 ( Figura 3 ).

**Figura 4:**
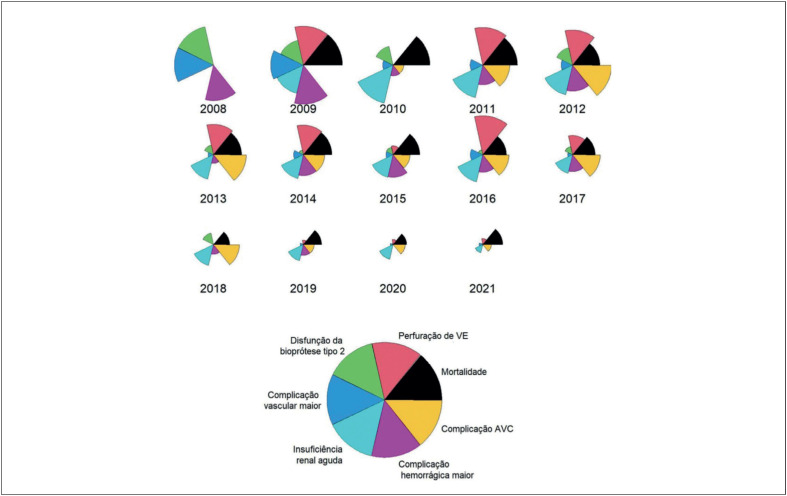
Gráfico *star plot* das principais complicações ligadas a mortalidade a cada ano. AVC: acidente vascular cerebral, Disfunção da biopróteses tipo 2= insuficiência da bioprótese; VE: ventrículo esquerdo.

Na análise comparativa dos desfechos a cada ano realizada pelo *star plot,* observa-se, de modo geral, uma redução dos desfechos do estudo, tanto da mortalidade como das complicações ao longo do tempo ( Figura 4 e Tabela 2 suplementar ).

## Discussão

A média de mortalidade intra-hospitalar do RIBAC-NT pode ser considerada elevada para o período observado. Os registros nacionais de outros países apresentaram mortalidade de cerca de 10% nos primeiros anos de TAVI,^16^ com queda gradual a cada ano, atingindo valores entre 1,3% e 3,5% mais recentemente, com média de mortalidade de 2% a 3,5% na última década.^17 , 18^ Com um número cada vez maior de pacientes submetidos a TAVI a cada ano, os óbitos diminuíram em razão de melhora da técnica, evolução dos dispositivos e abordagem de pacientes menos complexos. Embora o RIBAC-NT demonstre claramente uma queda na mortalidade a cada ano, essa não atingiu o percentual dos registros de outros países citados na referência.

Uma hipótese para explicar a mortalidade elevada no RIBAC-NT é a característica de que somente os cardiologistas intervencionistas principiantes nessa tecnica seriam obrigados a registrar seus primeiros casos para adquirir a certificação no implante de bioprótese por cateter pela SBHCI, o que representa um registro com viés de início da curva de aprendizado. Essa mortalidade elevada pode ainda refletir a possível expansão do procedimento para centros de menor volume. Estudo recente sobre a influência da experiência do operador sobre os resultados intra-hospitalares de TAVI mostrou que esse não é um fator de interferência quando há eficácia do *heart team* hospitalar.^19^ Cabe ressaltar que Bestehorn et al.^20^ avaliaram a relação do volume de procedimentos do centro com os desfechos no registro alemão e encontraram uma contínua e significativa diminuição das complicações e da mortalidade com o aumento do volume dos centros, com 5,6% ± 5% de mortalidade em hospitais com volume anual < 50 TAVI e de 2,4% ± 1% naqueles com volume > 200 TAVI. A TAVI tem uma longa curva de aprendizado e, dos 266 centros participantes do RIBAC-NT, poucos podem ser considerados de alto volume comparado aos dos países europeus ou norte-americano.

No nosso estudo, os fatores mais importantes associados a mortalidade foram as complicações decorrentes do procedimento, sendo a CV maior a mais relevante. É grande a variabilidade do percentual de CV descritas na literatura. Sardar et al.^21^ reportaram para as CV uma faixa de 1,9% a 30,7%, incluindo CV maiores e menores. No estudo Partner,^3^ que incluiu as próteses BE 1G, a frequência foi de 15%. Meta-análise realizada por Rahhab et al.,^22^ com 14.308 pacientes, encontrou média de 7,71% de CV maiores em análise que incluiu as duas gerações de biopróteses. Uma importante observação dos estudos foi o pior desfecho a curto prazo quando CV maiores ocorreram, assim como a diminuição da ocorrência dessas complicações com dispositivos de menor perfil. A frequência de CV maiores em nosso estudo foi de 6%, refletindo um número satisfatório comparado ao das publicações citadas. No entanto, chama a atenção a mortalidade de 34% associada à sua ocorrência, sugerindo pouca efetividade na resolução desses eventos, o que pode estar relacionado à falta de experiência do operador e da interação de um *heart team* multiprofissional, como ocorre nos grandes centros. A importância da CV na mortalidade em um registro composto, em boa parte, por casos referentes à curva de aprendizado da técnica demonstra a necessidade de atenção dos instrutores não somente com o ensino do emprego do dispositivo, mas com o ensino do passo a passo de todo o procedimento.

A IRA foi a segunda variável selecionada pela técnica de *machine learning* como associada com mortalidade. Na literatura, a frequência de IRA varia de 8% a 58%, estando associada com mortalidade 4 a 6 vezes maior,^23^ conforme observado em nosso estudo. São múltiplas as razões para o desenvolvimento de IRA nesse grupo de pacientes, entre elas o uso de meios de contraste, hipotensão temporária por diminuição de débito cardíaco durante a liberação das biopróteses, embolização de debris de aterosclerose para as artérias renais, hipovolemias, hemorragias e regurgitação importante da prótese.^24^

Apesar de o percentual de óbitos nas mulheres ser superior ao nos homens, o gênero não foi associado com mortalidade em nenhum dos modelos estatísticos apresentados, à semelhança do que encontramos na literatura onde mulheres se beneficiam de TAVI.^10^

As complicações não fatais ocorrem com maior frequência nas biopróteses 1G, devido ao seu perfil elevado, às características dos dispositivos de implante com maior dificuldade técnica de manuseio e à menor experiência dos operadores e dos centros.^22 , 25 , 26^ A primeira bioprótese liberada para uso no Brasil foi a *AE CoreValve* , implantada em 56,6% dos pacientes com próteses 1G. Essas próteses apresentavam dificuldade de posicionamento na altura adequada, o que podia levar a contato indesejado com o sistema de condução, associando-se, dessa forma, com maior necessidade de implante de marca-passo definitivo e com novo bloqueio de ramo esquerdo,^6 , 25^ justificando o maior índice de complicação nos portadores de ritmo sinusal ou fibrilação/flutter atrial do que no grupo com ritmo prévio de marca-passo. As próteses BE 1G foram as únicas passíveis de implantação por acesso transapical, o que justifica a sua associação com complicações pelo acesso de via não femoral. Atualmente não há evidências robustas sobre a segurança e a eficácia desse acesso, sendo recomendado apenas em situações excepcionais.^27 , 28^

A maior frequência de complicações intra-hospitalares em mulheres com uso de próteses 2G foi um achado sem referência na literatura, mas pode ser justificada por alguns dados anatômicos característicos desse gênero. Por serem compatíveis com artérias femorais menos calibrosas, essas próteses passaram a atender um número maior de mulheres, especialmente as de menor porte, antes incompatíveis com implante transcateter. Porém, devido a vasos de menor diâmetro, mais CV e hemorrágicas ocorrem no gênero feminino,^28 - 30^ não sendo diferente no RIBAC-NT. A anatomia pequena do complexo valvar aórtico com coronárias baixas aumenta o risco de oclusão coronária, complicação associada a próteses BE que ocorreu 4 vezes mais em mulheres no RIBAC-NT. No maior registro multicêntrico reportando obstrução coronária pós-TAVI, mulheres apresentaram a maioria das ocorrências (> 80%).^31^ Ressalta-se finalmente que, como no RIBAC-NT, numerosos estudos clínicos sobre gênero demonstram que complicações do procedimento de TAVI são mais frequentes em mulheres, mas não se traduzem em maior mortalidade, havendo diversas explicações para esse fato nos estudos clínicos.^8 - 10^

### Limitações do estudo

Nosso estudo consistiu em uma análise retrospectiva realizada em banco de dados eletrônico construído voluntariamente. Por esse motivo, pode estar sujeito a viés resultante do aporte de dados por múltiplos cardiologistas intervencionistas, embora todas as variáveis fossem padronizadas e o aporte de dados orientado por manual de instrução. A falta de aporte de dados foi outra limitação importante, embora não tenha prejudicado a análise ( Figura 1 suplementar ). O aporte de dados obrigatório somente para os profissionais principiantes, sendo facultativo após obtida a certificação de proficiência no método, contribuiu para um percentual grande de aportes durante a curva de aprendizado. A anonimização dos dados impediu o conhecimento de diferenças regionais, entre centros de porte diferente e entre profissionais de graus diversos de experiência. Porém, esse é o único banco de dados sobre TAVI em nosso meio, constituindo a fonte que permite avaliar a incorporação dessa tecnologia no Brasil, sendo, portanto, de grande importância nesse campo.

## Conclusão

A análise do registro RIBAC-NT mostrou que a mortalidade e as complicações não fatais intra-hospitalares estão associadas principalmente ao procedimento e menos aos dados demográficos e às comorbidades. A análise também mostrou gradativa diminuição da mortalidade e das complicações não fatais intra-hospitalares com o decorrer dos anos de incorporação da tecnologia. O gênero feminino foi associado com complicações não fatais, mas não com mortalidade ( Figura Central ).

## * Material suplementar

Para tabelas suplementares, por favor, clique aqui .

Para figura suplementar, por favor, clique aqui .
