# Supplementary material for: Desfechos Intra-Hospitalares no Registro Brasileiro de Implante de Bioprótese Aórtica por Cateter – 14 Anos em Análise
Source: Arq Bras Cardiol. 2024 May 7;121(5):e20230551. [Article in Portuguese] doi: 10.36660/abc.20230551 (PMC11081308; doi:10.36660/abc.20230551)
Supplement: Translation

# Translation: In-Hospital Outcomes in the Brazilian Registry of Transcatheter Aortic Valve Implantation – 14-Year Analysis

**DOI:** 10.36660/abc.20230551i

**Authors:** Maria Cristina Meira Ferreira, Viviana de Mello Guzzo Lemke, Maria Sanali Moura de Oliveira Paiva, Emilia Matos do Nascimento, Basílio de Bragança Pereira, Gláucia Maria Moraes de Oliveira

**Affiliations:** 1 Rio de Janeiro RJ Brazil Universidade Federal do Rio de Janeiro, Rio de Janeiro, RJ – Brazil; 2 Curitiba PR Brazil Cardiocare Clinica Cardiológica, Curitiba, PR – Brazil; 3 Natal RN Brazil Instituto Atena de Pesquisa Clínica, Natal, RN – Brazil; 4 Rio de Janeiro RJ Brazil Universidade do Estado do Rio de Janeiro, Rio de Janeiro, RJ – Brazil

**Keywords:** Aortic Valve Disease, Aortic Valve Stenosis, Heart Valve Prosthesis Implantation, Transcatheter Aortic Valve Replacement, Heart Valve Prosthesis

## Abstract

**Background:**

Transcatheter aortic valve implantation (TAVI) has had an exponential increase of its indication, being incorporated into the Brazilian Unified Public Health System in 2022, thus requiring assessment of its use in Brazil.

**Objeticve:**

To assess the factors associated with in-hospital mortality and non-fatal complications in both genders in the Brazilian Registry of Transcatheter Aortic Valve Implantation and New Technologies (RIBAC-NT) population.

**Method:**

Analysis of the RIBAC-NT database from 2008 to 2022 was performed. Logistic models and machine learning were used for statistical assessment of the association between variables and outcomes. The software R was used and a 5% significance level, adopted.

**Results:**

Analysis of 2588 patients (women, 51.2%; in-hospital death, 8.2%). Mortality was associated with procedural complications, of which major vascular complication (VC) and acute kidney injury (AKI) stood out (p<0.001). Major VC occurred in 6% of the patients, with 34% mortality; AKI occurred in 8.8%, with 13% mortality, which increased up to 8 times when AKI coexisted with other complications. Non-fatal complications occurred in 50.5% of all patients, affecting 63% of those with 1^st^generation (1G) bioprosthesis and 39% of those with 2^nd^generation (2G) bioprosthesis (p<0,001). Non-femoral access and heart rhythm influenced non-fatal complications in patients with 1G prostheses, while complications in patients with 2G prosthesis associated with the female gender (39.6% vs. 30.4%, p=0.003).

**Conclusion:**

In-hospital mortality in the RIBAC-NT population was directly associated with procedural complications, mainly major VC and AKI. The occurrence of non-fatal complications differed according to gender and bioprosthesis type.

## Introduction

Degenerative aortic stenosis, which currently afects 2% to 5% of adults over 65 years of age, has become a public health problem worldwide in recent decades.^1^In Brazil, the prevalence of calcific aortic valve disease increased by 201.8% from 1990 to 2019, reflecting population aging.^2^Transcatheter aortic valve implantation (TAVI) has appeared as an option to reduce mortality in patients with aortic stenosis in whom surgery would be difficult or risky.^3 - 5^Although several clinical studies have played an important role in the TAVI procedure evolution, a recent publication by Barili et al.,^6^analysing 8 randomized clinical trials on TAVI *versus* surgical aortic valve replacement, has identified the occcurrence of several biases with a systematic selective imbalance, favoring TAVI, such as deviation from random assigned treatment and loss to follow-up 6.7 times and 2.5 times smaller, respectively, in the TAVI group, while additional treatments during the procedure were 3.7 times more frequent in the surgical group.

Aiming to assess and monitor the use of TAVI, several nationally-representative registries have been implemented in several countries, showing higher rates of mortality and of non-fatal complications as compared to those reported in clinical trials.^7^To better understand the results of the use of the TAVI technology in Brazil, the Brazilian Association of Interventional Cardiology (SBHCI) has implemented a registry of patients submitted to TAVI in Brazil, known as the Brazilian Registry of Transcatheter Aortic Valve Implantation and New Technologies (RIBAC-NT), which compiles data from patients undergoing TAVI since 2008.

The incorporation of the TAVI procedure into the Brazilian Unified Public Health System (SUS) in recent years requires follow-up and performance assessment regarding procedural complications in the different clinical scenarios in Brazil. Women represent a challenge, considering that, as compared to men, they have longer life expectancy, being, thus, more likely to undergo TAVI, and have more procedural complications, although without higher mortality.^8 - 10^Thus, this study was aimed at assessing, in patients from the RIBAC-NT submitted to TAVI, not only the factors associated with the outcomes in-hospital mortality and non-fatal complications, but the existence of an association between gender and those outcomes as well.

## Material and Methods

Observational, retrospective study analyzing the database from the RIBAC-NT from January 2008 to January 2022. The participation in the RIBAC-NT, which includes 266 centers in 20 Brazilian Federative Units, is voluntary. This study was submitted to and approved by the Committee on Ethics and Research on 09/21/2022 (CAAE 60808622.4.0000.5252).

The bioprostheses used in the RIBAC-NT were classified according to their generation and release mechanism ( supplemental Table 1 ).

The complications from the procedure were categorized according to the Valve Academic Research Consortium 2012 (VARC-2).^11^In this study, the bleeding events classified in VARC-2 as major or life threatening were grouped under the name of major bleeding complications.

### Statistical analysis

The population characteristics were described with dichotomous qualitative variables, presented as percentage of occurrence, and with continuous variables, presented as median and interquartile range. The chi-square test was used to compare the percentages of occurrence of events. The Shapiro-Wilk normality test rejected the hypothesis of normality of the variables. The non-normal data distribution was confirmed by use of graphic inspection.

The analysis of the factors associated with the outcomes mortality and non-fatal complications involved demographic, clinical and laboratory variables, and comorbidities, as well as the procedure's technical data and complications.

Initially the logistic regression model with elastic net regularization, a method to previously select the independent variables, was used.^12^Then, other logistic models were used, removing the variables that showed no statistical significance in the previous model. The use of different models was aimed at identifying patterns and associations that cannot be easily detected with only one model because of data complexity.

Machine learning techniques were used for the nonparametric models based on classification trees.^13^A hierarchy structure based on algorhythms was built, in the search for associations and interdependence of variables in the nodes.

The multinomial model was used to analyze the combined outcomes of death and non-fatal complications.

The Partykit package in R was used in the analysis.^14 , 15^The statistical significance level adopted was 5%.

A temporal analysis of the mortality and its related complications across the years studied was performed using star plot, a data visualization technique for multivariate analysis. The method produces star-shaped graphs, where each variable is represented as an area proportional to the value observed. The data used in the analysis resulted from the division of the values corresponding to the variables with higher impact on the outcomes from 2008 to 2021.^16^

## Results

The TAVI procedure was performed in 3793 patients (1883 men and 1910 women), 1205 of whom were excluded from the analysis due to lack of some information (619 men and 586 women, p= 0,067). Complete data were obtained from 2588 patients, who comprised our sample for analysis. No statistically significant difference was observed between genders ( supplemental Figure 1 ).

In-hospital death occurred in 8.2% of the patients, being proportionaly higher in women. Patients who died were older and study population was represented mostly by functional class III or IV. Chronic obstructive pulmonary disease (COPD) was the most commonly found comorbidity among those who died. Creatinine clearance was higher among those who survived (p= 0.0001), while the Society of Thoracic Surgeons (STS) score was higher among those who died (p<0.0001) ( Table 1 ).

**Table 1:** Occurrence rate of the demographic, clinical and laboratory variables, and comorbidities in patients of the RIBAC-NT, comparison between patients who survived and died

		Total	Survivors	Death
**Number of patients, n (%)**		2588 (100%)	2375 (91.8%)	213 (8.2%)
**Demographic data**				
	Men,n (%)	1264 (48.8%)	1181 (93.4%)	83 (6.6%)
	Women, n (%)	1324 (51.2%)	1194(90.2%)	130 (9.8%)
	Age, median (IQR)	82 (78—86)	82 (77—86)	84 (79—88)
	BMI, kg/m^2^, median (IQR)	25.8 (23.3—29.0)	25.8 (23.3—29)	26.2 (23.1—29.4)
**Clinical data**				
	Functional class III or IV, n (%)	1924 (74.3%)	495 (88.6%)	64 (11.4%)
	Angina pectoris, n (%)	637 (24.6%)	594 (93.2%)	43 (6.8%)
	Syncope, n (%)	681 (26.3%)	631 (92.7%)	50 (7.3%)
**Comorbidities**				
	CAD, n (%)	1399 (54%)	1284 (91.8%)	115 (8.2%)
	Previous AMI, n (%)	363 (14%)	332 (91.5%)	31 (8.5%)
	Previous stroke, n (%)	207 (7.9%)	188 (90.8%)	19 (9.2%)
	Carotid and cerebrovascular disease, n (%)	421 (16.2%)	383 (91.0%)	38 (9.0%)
	COPD, n (%)	474 (18.3%)	418 (88.2%)	56 (11.8%)
	Aortic aneurysm, n (%)	108 (4%)	99 (91.7%)	9 (8.3%)
	Diabetes mellitus, n (%)	869 (33.6%)	800 (92.1%)	69 (7.9%)
	Dyslipidemia, n (%)	1.495 (57.8%)	1.375(92.0%)	120 (8.0%)
	SAH, n (%)	2.148 (82.9%)	1966 (91.5%)	182 (8.5%)
	PAD, n (%)	493 (19%)	448 (90.9%)	45 (9.1%)
	PAH, n (%)	607 (23.5%)	547 (90.1%)	60 (9.9%)
	CKF, n (%)	1.917 (74%)	1.748 (91.2%)	169 (8.8%)
**Laboratory data**				
	Sinus rhythm, n (%)	2043 (78.9%)	1883 (92.2%)	160 (7.8%)
	Pacemaker rhythm, n (%)	214 (8.3%)	199 (93.0%)	15 (7.0%)
	Atrial fibrillation/Flutter, n (%)	331 (12.8%)	293 (88.5%)	38 (11.5%)
	Creatinine (mg/dL), median (IQR)	1.1 (0.9—1.4)	1.1 (0.9—1.4)	1.1 (0.9—1.6)
	Creatinine clearance (mL/min), median (IQR)	46.6 (34.8—62.2)	47 (35.2—62.4)	39.5 (29.8—57.4)
	EF echocardiogram, (%), median (IQR)	63 (53—69)	63 (53—69)	63 (50—69)
	STS score, median (IQR)	5.5 (3.4—10.2)	5.3 (3.3—9.9)	7.7 (4.5—14.0)

AMI: acute myocardial infarction; BMI: body mass index; CAD: coronary artery disease; CKF: chronic kidney failure; COPD: chronic obstructive pulmonary disease; EF: ejection fraction; IQR: interquartile range; PAD: peripheral artery disease; PAH: pulmonary arterial hypertension; SAH: systemic arterial hypertension; STS: Society of Thoracic Surgeons.

The logistic model identified the variables COPD, creatinine level, and procedural complications attributable to the bioprosthesis, such as clinical, surgical and mechanical, as those associated with death ( Table 2 ).

**Table 2:** Variables identified in the logistic regression model as associated with the outcome death

	Estimate	Std. Error	z value	Pr(>|z|)	
Creatinine	0.25226	0.07389	3.414	0.000640	***
COPD	0.67307	0.18777	3.585	0.000338	***
Complication: AMI	2.03395	0.53088	3.831	0.000127	***
Complication: stroke	1.81760	0.32690	5.560	2.70e-08	***
Major bleeding complication	0.91282	0.23560	3.874	0.000107	***
Minor bleeding complication	-0.04292	0.51031	0.084	0.932966	
Acute kidney injury	1.40844	0.17848	7.891	2.99e-15	***
Major vascular complication	1.25127	0.26325	4.753	2.00e-06	***
Minor vascular complication	-0.50648	0.49991	-1.013	0.310990	
Bioprosthesis stenosis	12.15345	535.41122	-0.023	0.981890	
Bioprosthesis regurgitation	1.34886	0.41188	3.275	0.001057	**
Left bundle branch block	-0.58469	0.26618	-2.197	0.028047	*
Need for PPM	-0.93291	0.32887	-2.837	0.004558	**
Coronary obstruction	3.77983	1.23676	3.056	0.002241	**
VSD	1.94111	1.14184	1.700	0.089135	
LV perforation	2.09158	0.51046	4.097	4.18e-05	***

Statistical significance

‘***’ 0.001,

‘**’ 0.01,

‘*’ 0.05,

‘ ’1. Std. Error = standard error, z value = percentile of the standard normal distribution, Pr(>|z|) = significance level. AMI: acute myocardial infarction; COPD: chronic obstructive pulmonary disease; LV: left ventricular; PPM: permanent pacemaker; VSD: ventricular septal defect.a

The classification tree built from the variables selected in the logistic model showed the association between procedural complications and death. The first variable selected was major vascular complication (VC) (node 1), followed by acute kidney injury (AKI) (node 2). In the absence of those complications, mortality was 4.3% (node 3) ( Figure 1 ).

**Figure 1:** Classification tree of in-hospital mortality related factors. AKI= acute kidney injury, LV= left ventricle.

Patients with major VC associated with left ventricular (LV) perforation had a mortality of 73.3% (nodes 1, 11, and 13), and in the absence of LV perforation, mortality was 34.8% (nodes 1 and 12). When VC was absent or minor type, and in the presence of AKI, mortality varied according to the coexistence of stroke, with mortality of 84.6% (nodes 2, 4, and 10); bioprosthesis dysfunction of the regurgitation type, mortality of 58.3% (nodes 2, 5, and 9); major bleeding complication, mortality of 31.7% (nodes 2, 6, and 8); and minor bleeding complication, mortality of 13% (nodes 2, 6, and 7) ( Figure 1 ).

The statistical models did not identify gender as a factor associated with mortality, although complications predominated in the female gender. Major VC occurred in 156 (6%) patients, more frequently in women (8.1% *vs* . 3.9%, p< 0.001), LV perforation occurred in 25 (0.9%), twice more frequently in the female gender (1.4% *vs* . 0.6%, p= 0.03), and minor bleeding complication occurred in 87 (3.7%), mainly in women (5.3% *vs* . 3.2%, p=0.05). Acute kidney injury was observed in 355 (13.7%) patients and stroke, in 65 (2.5%), both without difference between genders. Bioprosthesis dysfunction of the regurgitation type occurred in 47 (1.8%) patients, more often in men (2.4% *vs.* 1.3%, p=0.03), while that of the stenosis type occurred in only 1 patient in the study.

Regarding the non-fatal complications, 1308 (50.5%) patients had some type of complication related to the procedure. Complications occurred more frequently with the 1st generation (1G) bioprostheses, which were used in 47.3% of the study population, as compared to the 2nd generation (2G) ones, affecting 774 (63.1%) patients *versus* 532 (39.1%), respectively, p<0.001.

Of the independent variables with statiscally significant association with non-fatal complications, the logistic regression model identified female gender, previous stroke and pacemaker rhythm, as well as bioprosthesis type ( Table 3 ).

**Table 3:** Variables identified in the logistic regression model as associated with the outcome non-fatal complications

	Estimate	Std.Error	z value	Pr(>|z|)	
Women	0.20839	0.08689	2.398	0.01647	*
History of stroke	0.52861	0.16235	3.256	0.00113	**
PPM rhythm	-0.74091	0.16631	-4.455	8.39e-06	***
AF/Atrial flutter rhythm	-0.13150	0.13223	-0.99	0.32001	
Non-femoral vascular access	0.38136	0.19996	1.907	0.05650	
Self-expanding bioprosthesis	-0.64416	0.08935	-7.210	5.61e-13	***
ME bioprosthesis	0.21087	0.28060	0.751	0.45236	
2^nd^generation bioprosthesis	-0.94258	0.08841	-10.661	< 2e-16	***

Statistical significance:

‘***’ 0.001,

‘**’ 0.01,

‘*’ 0.05,

‘ ’1. Std. Error = standard error, z value = percentile of the standard normal distribution, Pr(>|z|) = significance level. AF: atrial fibrillation; ME: mechanically expanded; PPM: permanent pacemaker.

The classification tree built from the variables selected in the logistic model showed that the non-fatal complications differed according to the generation and release mechanism of the bioprosthesis used. Node 1 divides the tree into two branches according to the bioprosthesis generation. The 1G prostheses had different association with complications depending on the release mechanism (node 2). When self-expanding (SE) or mechanically expanded (ME) bioprostheses were used, the percentage of complications differed depending on heart rhythm (node 3), being 70.3% in patients with sinus rhythm or atrial fibrillation/flutter (node 4) and 42.4% in patients with pacemaker rhythm (node 5). The complications of the balloon-expandable (BE) 1G prostheses showed association with the vascular access used (node 6). With the femoral access, the complication rate was 41.5% (node 7), while with the non-femoral access, 63.6% (node 8) ( Figure 2 ).

**Figure 2:** Classification tree of non-fatal related factors. AF: atrial fibrillation.

When 2G prostheses were used, the occurrence of complications associated with gender (node 9), being lower in the male gender (30.4% - node 10) than in the female gender (39.6% - node 11) ( Figure 2 ).

The multinomial model used in the analysis of the combined outcomes death and non-fatal complications showed associations between pacemaker rhythm and non-fatal complications, between COPD and death, and between the use of BE prostheses and non-fatal and fatal complications ( Table 4 ).

**Table 4:** Variables identified in the logistic regression model as associated with the outcomes non-fatal and fatal complications

	Estimate	Std. Error	z value	Pr(>|z|)	
**NON-FATAL COMPLICATIONS**					
Creatinine	-0.071772	0.057691	-1.2441	0.2134779	
Angina	-0.120351	0.097514	-1.2342	0.2171300	
COPD	0.010330	0.110159	0.0938	0.9252862	
Pacemaker rhythm	-0.672786	0.161549	-4.1646	3.119e-05	***
AF/atrial flutter rhythm	-0.103293	0.128402	-0.8045	0.4211338	
BE bioprosthesis	-0.668686	0.086679	-7.7145	1.221e-14	***
ME bioprosthesis6	0.047406	0.516099	0.0919	0.9268136	
**FATAL COMPLICATIONS**					
Creatinine	0.121578	0.072942	1.6668	0.0955582	
Angina	-0.357984	0.184781	-1.9373	0.0527033	
COPD	0.495042	0.174086	2.8437	0.0044598	**
Pacemaker rhythm	-0.395878	0.286658	-1.3810	0.1672751	
AF/atrial flutter rhythm	0.298336	0.201967	1.4771	0.1396355	
BE bioprosthesis	-0.523401	0.155061	-3.3755	0.0007369	***
ME bioprosthesis	0.047406	0.516099	0.0919	0.9268136	

Statistical significance:

‘***’ 0.001,

‘**’ 0.01,

‘*’ 0.05,

‘ ’1. Std. Error = standard error, z value = percentile of the standard normal distribution, Pr(>|z|) = significance level. AF: atrial fibrillation; BE: balloon-expandable; COPD: chronic obstructive pulmonary disease; ME: mechanically expanded.

The classification tree built from the variables selected in the elastic net of multinomial modeling shows that the release mechanism of the prosthesis (node 1) divides the tree into two branches. In patients who received SE and ME prostheses, the complications differed according to the previous heart rhythm (node 2). With sinus or atrial fibrillation/flutter rhythm, non-fatal complications occurred in 50% of the patients, while fatal complications occurred in 9% (node 3). In patients with previous pacemaker rhythm, non-fatal complications occurred in 31.1%, while fatal complications, in 7.4% (node 4). These data support the analysis of the previous estatistical model. In patients with BE prostheses, non-fatal complications occurred in 33.7%, while fatal complications, in 7.3% (node 5), regardless of the heart rhythm. The frequency of non-fatal complications was higher in patients in node 3, but the mortality rates were similar in nodes 3, 4, and 5 ( Figure 3 ).

**Figure 3:** Classification tree: compost outcomes in-hospital death and non-fatal complications.

In the comparative analysis of the outcomes performed yearly by use of the star plot, a reduction in the study outcomes, both mortality and complications, was observed over time ( Figure 4 and supplemental Table 2 ).

**Figure 4:** Star plot graph of the main complications associated with mortality per year. Bioprosthesis dysfunction type 2= insufficiency; LV: left ventricular.

## Discussion

The mean in-hospital mortality in the RIBAC-NT can be considered elevated for the period studied. The registries of other countries had mortality around 10% in the initial years of the TAVI procedure implementation.^16^There has been a gradual decline per year, reaching figures between 1.3% and 3.5% in most recent years, with mean mortality ranging from 2% to 3.5% in the past decade.^17 , 18^With the growing number of patients undergoing TAVI annually, the number of deaths has decreased due to technique improvement, evolution of the devices, and treatment of less complex patients. Despite the clear mortality reduction observed in the RIBAC-NT over the years, the figures of the registries of the other countries have not been reached.

One hypothesis for the high mortality in the RIBAC-NT is that the entry of TAVI procedure data into that registry is mandatory only for interventional cardiologists at the beginning of their TAVI practices to obtain their SBHCI transcatheter prosthesis implantation certification. Thus, that high mortality might be explained not only by a learning curve bias but also by the possible expansion of the procedure to lower-volume centers. A recent study on the influence of the operator's experience on the in-hospital results of TAVI procedures has shown that, in the presence of an effective heart team, that is not a factor of interference.^19^It is worth noting that Besterhorn et al.,^20^assessing the center's volume-outcome relationship in the German registry, have found a continuous and statistically significant reduction in complications and mortality associated with the increasing TAVI volume of a center, with mortality of 5.6% ± 5% in hospitals with an annual volume < 50 TAVI and of 2.4% ± 1% in those with an annual volume > 200 TAVI. The TAVI procedure has a long learning curve and, of the 266 centers participating in the RIBAC-NT, only a few can be considered high-volume as compared to those in European and North American countries.

In our study, the most important factors associated with mortality were procedural complications, major VC being the most relevant. The percentage of VCs reported in the literature is high. Sardar et al.^21^have reported VC frequency ranging from 1.9% to 30.7%, including major and minor VCs. In the Partner Trial,^3^which included BE 1G prostheses, that frequency was 15%. A meta-analysis conducted by Rahhab et al.,^22^with 14 308 patients, has found a mean of 7.71% of major VCs in an analysis including the two generations of bioprostheses. An important finding of the studies was worse short-term outcomes when major VCs occurred and a reduction in the occurrence of those complications with low-profile devices. In our study, the frequency of major VCs was 6%, which is satisfactory when compared to that of the mentioned studies. However, the 34% mortality associated with their occurrence found in our study stands out, suggesting low effectiveness in solving those events, which might be related to the operator's lack of experience and the lack of the multiprofessional heart team interaction. The importance of VC to mortality in a registry comprising mainly data from the interventional cardiologists’ learning curves of TAVI shows the need for supervision regarding guidance not only on the device's use, but also during all the procedure, step by step.

Acute kidney injury was the second variable associated with mortality selected in the machine learning technique. In the literature, the frequency of AKI ranges from 8% to 58%, being associated with mortality 4 to 6 times higher,^23^as observed in our study. The development of AKI in that group of patients has multiple reasons, such as the use of contrast media, transient hypotension due to output decrease during bioprosthesis release, atherosclerotic debris embolization to renal arteries, hypovolemia, bleeding, and important prosthesis regurgitation.^24^

Although the percentage of women's deaths is higher than men's, gender was not associated with mortality in any of the statistical models used, similarly to that found in the literature where women benefit from TAVI.^10^

Non-fatal complications most frequently occur with 1G bioprostheses, because of their high profile, higher technical difficulty for their handling, as well as the little experience of the operators and centers.^22 , 25 , 26^The first bioprosthesis available for use in Brazil was the *AE CoreValve* , implanted in 56.6% of the patients with 1G prostheses. One challenge of those prostheses was the difficulty in positioning at the proper height, which could lead to unwanted contact with the conduction system and, thus, higher need for a permanent pacemaker implantation, in addition to a new left bundle branch block,^6 , 25^justifying the higher rate of complication in patients with sinus rhythm or atrial fibrillation/flutter than in the group with previous pacemaker rhythm. The BE 1G prostheses were the only ones that could be implanted through transapical access, justifying their association with complications related to the non-transfemoral access. Currently, there is no firm evidence of the safety and efficacy of that access site, being only exceptionally recommended.^27 , 28^

Our finding of higher frequency of in-hospital complications in women with the use of 2G prostheses has not been reported in the literature, but can be justified by some anatomical characteristics of the female gender. By being compatible with femoral arteries of smaller caliber, those prostheses have met the need of a larger number of women, mainly the small ones, who were formerly unsuitable for transcatheter implantation. However, because of vessels of smaller diameters, more vascular and bleeding complicatins occur in the female gender,^28 - 30^which was not different in the RIBAC-NT. The small size of the valvar aortic complex with low coronary arteries increases the risk of coronary obstruction, a complication associated with BE prostheses that occurred 4 times more often in women in the RIBAC-NT. In the largest multicenter registry reporting post-TAVI coronary obstruction, most of the cases were observed in women (> 80%).^31^It is worth noting that, similarly to the RIBAC-NT, numerous clinical trials on gender have shown that TAVI procedural complications are more frequent in women, but do not translate into higher mortality, and the clinical trials have provided several explanations for that.^8 - 10^

### Study limitations

Our study was a retrospective analysis of an electronic database, in which participation is voluntary. Thus, bias regarding data input by multiple interventional cardiologists might have occurred, although all variables had been standardized and data input guided by an instruction manual. In addition, lack of data input was another important limitation, even though that has not hindered data analysis ( supplemental Figure 1 ). The fact that the entry of TAVI procedure data into the RIBAC-NT is mandatory only for interventional cardiologists at the beginning of their practices to obtain their SBHCI certification, but not after that, contributes to a high percentage of registry entries during the interventional cardiologists’ learning curves. Data anonymization hindered knowledge of the differences between regions, between centers of different volumes, and between professionals of different experience levels. However, that is the only database on the TAVI procedure in Brazil, a source for assessing the incorporation of that technology, thus, a highly important registry.

## Conclusion

The analysis of the RIBAC-NT registry showed that in-hospital mortality and non-fatal complications are mainly associated with the procedure and less associated with demographic data and comorbidities. In addition, the analysis showed a gradual reduction in in-hospital mortality and non-fatal complications over the years of the TAVI technology incorporation. Female gender associated with non-fatal complications, but not with mortality ( Central Illustration ).

## *Supplemental Materials

For supplemental tables, please click here .

For supplemental figure, please click here .
